# Modeling Cancer Cell Growth Dynamics *In vitro* in Response to Antimitotic Drug Treatment

**DOI:** 10.3389/fonc.2017.00189

**Published:** 2017-08-30

**Authors:** Alexander Lorz, Dana-Adriana Botesteanu, Doron Levy

**Affiliations:** ^1^CEMSE Division, King Abdullah University of Science and Technology (KAUST), Thuwal, Saudi Arabia; ^2^Sorbonne Universités, UPMC Univ Paris 06, UMR 7598, Laboratoire Jacques-Louis Lions, Paris, France; ^3^Women’s Malignancies Branch, Center for Cancer Research, National Cancer Institute, Bethesda, MD, United States; ^4^Department of Mathematics and Center for Scientific Computation and Mathematical Modeling (CSCAMM), University of Maryland, College Park, MD, United States

**Keywords:** apoptosis, cell-cycle variations, intrinsic heterogeneity, mitotic arrest, OVCAR-8, partial differential equations, population dynamics

## Abstract

Investigating the role of intrinsic cell heterogeneity emerging from variations in cell-cycle parameters and apoptosis is a crucial step toward better informing drug administration. Antimitotic agents, widely used in chemotherapy, target exclusively proliferative cells and commonly induce a prolonged mitotic arrest followed by cell death via apoptosis. In this paper, we developed a physiologically motivated mathematical framework for describing cancer cell growth dynamics that incorporates the intrinsic heterogeneity in the time individual cells spend in the cell-cycle and apoptosis process. More precisely, our model comprises two age-structured partial differential equations for the proliferative and apoptotic cell compartments and one ordinary differential equation for the quiescent compartment. To reflect the intrinsic cell heterogeneity that governs the growth dynamics, proliferative and apoptotic cells are structured in “age,” i.e., the amount of time remaining to be spent in each respective compartment. In our model, we considered an antimitotic drug whose effect on the cellular dynamics is to induce mitotic arrest, extending the average cell-cycle length. The prolonged mitotic arrest induced by the drug can trigger apoptosis if the time a cell will spend in the cell cycle is greater than the mitotic arrest threshold. We studied the drug’s effect on the long-term cancer cell growth dynamics using different durations of prolonged mitotic arrest induced by the drug. Our numerical simulations suggest that at confluence and in the absence of the drug, quiescence is the long-term asymptotic behavior emerging from the cancer cell growth dynamics. This pattern is maintained in the presence of small increases in the average cell-cycle length. However, intermediate increases in cell-cycle length markedly decrease the total number of cells and can drive the cancer population to extinction. Intriguingly, a large “switch-on/switch-off” increase in the average cell-cycle length maintains an active cell population in the long term, with oscillating numbers of proliferative cells and a relatively constant quiescent cell number.

## 1 Introduction

Intratumoral cancer heterogeneity represents a major obstacle to improving the overall response and survival of cancer patients (1–4). While most tumors initially respond well to drug therapies, many will relapse at a certain point following treatment (5, 6). One of the major reasons behind therapeutic failure is attributed to cancer cell-intrinsic factors, such as variations in cell-cycle parameters (e.g., cell-cycle duration, apoptosis length, mitotic index, percentage of apoptotic cells) and the presence of quiescent cancer cells, both of which decrease the efficacy of therapies that rely on active cell cycling (7–10).

Antimitotic cancer drugs represent a highly diverse and successful class of antimitotic agents, reported to have a broad spectrum of potent anti-tumor activity in various hematological and solid malignancies (7, 11–17). Examples of such drugs include microtubule-targeting agents, e.g., taxanes and vinca alkaloids, and newer agents that disrupt mitosis without affecting microtubule dynamics, e.g., kinesin spindle protein inhibitors and inhibitors of mitotic kinases (18–28).

While the primary drug target depends on the antimitotic agent used, pre-clinical data from in vitro experiments showed that prolonged mitotic arrest occurs in 100% of the cell populations under study irrespective of the agent used (29–33). However, these data also revealed that while all proliferating cells will undergo mitotic arrest when exposed to high concentrations of antimitotic drugs, there is considerable cell-to-cell variation of apoptotic response to antimitotic drugs in human cancer cell lines. Such observations have been reported in multiple single-cell studies involving individual cancer cells in culture in the presence of various antimitotic drugs, including kinesin-5 inhibitors (30, 32) taxol (29, 31–37), and nocodazole (32, 38–40). In the presence of identical drug exposure times and concentrations, the extent of heterogeneity in cellular response reported both within and across cancer cell lines is considerable (29–33, 35–37). For example, in Ref. (32), the authors analyzed 15 different cancer cell lines for their long-term response to different antimitotic drugs. They found that cellular responses to identical drugs are heterogeneous, e.g., within each distinct cell line, cells exhibit different responses following prolonged mitotic arrest, such as undergoing apoptosis after exiting mitosis, dying after completing several mitoses, or dying in interphase.

Investigating the role of intrinsic cell heterogeneity emerging from variations in cell-cycle parameters and apoptosis in cancer cell growth dynamics *in vitro* is a crucial first step toward better informing antimitotic drug administration. Several mathematical models have been formulated to investigate the dynamic variations among different cellular phenotypes and their role in the emergence of adaptive evolution and chemotherapeutic resistance (41–45) or the impact of cancer cell size, age, and cell-cycle phase in predicting the long-term *in vitro* population growth dynamics (46–55).

For example, in Ref. (46), the authors modeled the cancer cell population dynamics using a system of four partial differential equations (PDEs) representing the four cell-cycle phases (i.e., *G*_0_, *G*_1_, *S*, and *M*) with relative DNA content as the structuring variable. The goal therein was to obtain the steady DNA distributions for each cell-cycle phase and match the flow cytometry DNA profiles of the human melanoma NZM13 cell line at various time points following the addition of paclitaxel.

In Ref. (48), the authors derived two novel mathematical models, a stochastic agent-based model and an integro-differential equation model, in order to study the effect of cell-cycle-induced intrinsic tumor heterogeneity on the overall growth dynamics. Both models characterized the growth of cancer cells as dynamic interactions between the proliferative, quiescent, and apoptotic states. The models were designed to predict the cancer growth as a function of the intrinsic heterogeneity in the duration of the cell-cycle and apoptosis process and also included cellular density dependency effects. An extension of these models to spatial models was done in Ref. (49).

In this paper, we reformulated the models of Ref. (48). Specifically, we assumed that cells are structured by their age, i.e., how long each cell will spend in the cell cycle or apoptosis. The advantages of the present approach lie in the ability to access directly the cellular age in each compartment and to study the impact of prolonged mitotic arrest induced by antimitotic agents on the long-term population growth dynamics. Our model comprises of two PDEs for the proliferative and apoptotic cell compartments structured in cellular age and one ordinary differential equation for the quiescent compartment. We modeled the prolonged mitotic arrest induced by the drug as an increase in the average cell-cycle length duration, a consequence of the slowing or blocking of mitosis at the metaphase-anaphase transition (30, 34, 38, 56). We assumed that if the total time a cell spends in the cell cycle is greater than the cell-cycle age threshold, apoptotic cell death is triggered, a phenomenon observed *in vitro* (18, 30, 33, 34, 37, 38, 56–61). We used numerical simulations to subsequently study the impact of increasing the cell-cycle length on the overall population survival.

Our results suggest that at confluence and in the absence of any drug, quiescence is the long-term asymptotic behavior emerging from the cancer cell growth dynamics. This pattern is maintained in the presence of a small increase in the average cell-cycle length. However, an intermediate increase in cell-cycle length markedly decreases the total number of cancer cells present and can drive the cell population to extinction. A large “switch-on/switch-off” increase in the average cell-cycle length maintains an active cell population in the long term, with oscillating numbers of proliferative cells and a relatively constant quiescent cell number. Intriguingly, our results suggest that a large “switch-on/switch-off” increase in the average cell-cycle length may maintain an active cancer cell population in the long term.

This work is aimed at understanding cancer cell growth dynamics in the context of cancer heterogeneity emerging from variations in cell-cycle and apoptosis parameters. The mathematical modeling framework proposed herein merits consideration as one of the few mathematical models to investigate dynamic cancer cell responses to prolonged mitotic arrest induced by antimitotic drug exposure. Our proposed modeling framework can serve as a basis for future studies of the heterogeneity observed *in vitro* of cancer cell responses in the presence of antimitotic drugs.

## 2 Materials and Methods

### 2.1 Model Setup

**Figure 1:**
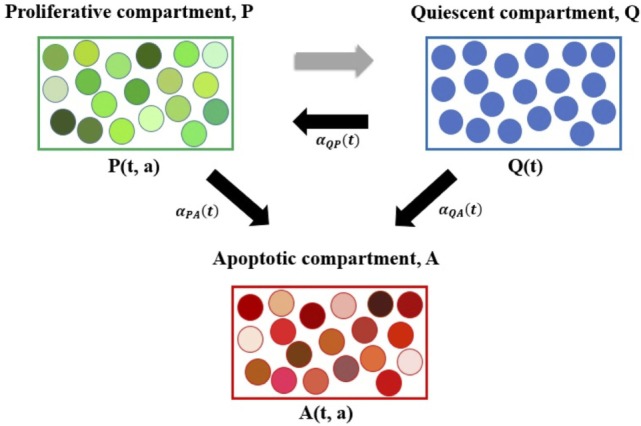
Diagram representing the age-structured mathematical modeling framework. Here, *P* denotes the proliferative compartment, with *P*(*t*, *a*) cells present at time *t* with time *a* remaining to be spent in this compartment. Proliferative cells can either transition to *A* or to *Q* at *a* = 0 upon completion of the cell cycle. *Q* denotes the quiescent compartment, with *Q*(*t*) cells present at time *t*. Quiescent cells can either transition to *P* with rate *α_QP_*(*t*) or to *A* with rate *α_QA_*(*t*). *A* denotes the apoptotic compartment, with *A*(*t*, *a*) cells present at time *t* and time *a* remaining to be spent in this compartment before completing apoptosis. For illustration purposes, cells within each compartment are grouped together. The various shades of green represent the different times remaining to be spent by cells in the proliferative compartment (i.e., in the cell cycle) before transitioning. Similarly, the various shades of red represent the different times remaining to be spent by cells in the apoptotic compartment, before completing apoptosis and being removed from the numerical simulations. The three explicit transition rates (i.e., *α_QP_*(*t*), *α_PA_*(*t*), and *α_QA_*(*t*)) are illustrated using black arrows pointing in the direction of the respective transition. The implicit transition from *P* to *Q* representing the successful completion of the cell cycle is denoted by a gray arrow.

The system (1)–(3) is a novel physiologically motivated mathematical model that assumes continuous distributions on cellular age, i.e., the times spent in the cell-cycle and apoptosis process. The model consists of proliferative (i.e., cells actively dividing, in either a *G*_1_, *G*_2_ or *M*-like state), quiescent (i.e., a *G*_0_-like state), and apoptotic compartments, as illustrated in Figure 1.

The proliferative compartment is structured by the time remaining to be spent by cells in the cell cycle before successfully completing mitosis and doubling. The apoptotic compartment is structured by the time remaining for cells to fully degrade and complete apoptosis. Accordingly, the dynamics of the cancer cell population is governed by the following system:

(1) $$\partial_{t}P\left(t,a\right)-\partial_{a}P\left(t,a\right)=\alpha_{\mathrm{QP}}\left(t\right)Q\left(t\right)f_{P}\left(a\right)1_{\left[0,\bar{a}\right]}-\alpha_{\mathrm{PA}}\left(t\right)P\left(t,a\right),$$

(2) $$\partial_{t}Q\left(t\right)=2P\left(t,0\right)-\left(\alpha_{\mathrm{QP}}\left(t\right)+\alpha_{\mathrm{QA}}\left(t\right)\right)Q\left(t\right),$$

$$\begin{array}{llll} \partial_{t}A\left(t,a\right)-\partial_{a}A\left(t,a\right) & =\left[\alpha_{\mathrm{QA}}\left(t\right)Q\left(t\right)+\alpha_{\mathrm{PA}}\left(t\right)\int P\left(t,a\right)\;\mathrm{da}\right.\; & & \;\\ & \;\left.+\alpha_{\mathrm{QP}}\left(t\right)Q\left(t\right)\int f_{P}\left(a\right)1_{\left(\bar{a},\infty \right)}\;\mathrm{da}\right]\;f_{A}\left(a\right).\; & \text{(3)} \end{array}$$

Initial conditions for this system are described in the following.

### 2.2 Model Description

In these equations, *P*(*t*, *a*) represents the number of proliferative cells at time *t* that still spend *a* in this compartment before doubling. The rates of change of *P*(*t*, *a*) with respect to the experimental time course *t* and age *a* are represented by $\partial_{t}$ and $\partial_{a}$, respectively. The term $\partial_{a}$*P*(*t*, *a*) in equation (1) implies that the time remaining until proliferating cells complete the cell cycle decreases as time *t* advances.

When entering the cell cycle, each cell is assigned its individual amount of time to be spent cycling, i.e., *a*, which is randomly selected from the Gaussian distribution function with mean *μ* and SD *σ* and probability density function *f_P_*(*a*). When reaching *a* = 0, cells in *P* exit the cell cycle. The maximum length of time spent in *P* before exiting thus corresponds to the maximum length of the mitotic arrest induced by an antimitotic drug. We assumed that the transition of cells back to *Q* is due to a successful (i.e., non-aberrant) mitosis.

Cells in *Q* act as a reservoir for the other two compartments, i.e., they move into either the apoptotic or proliferative compartment with rates *α_QA_*(*t*) or *α_QP_*(*t*), respectively. Intuitively, quiescent cells do not actively progress through the cell cycle nor are committed to undergo apoptosis (i.e., they remain in a *G*_0_-like state).

Cells can undergo apoptosis immediately after exiting the cell cycle, after completing several mitoses, or during interphase. Once cells enter *A*, they are irreversibly committed to completing apoptosis and cannot transition back to either *P* or *Q*. When apoptosis is completed, cells are removed from the numerical simulation. The term $\partial_{a}$*A*(*t*, *a*) in equation (3) implies that the time remaining until cells complete apoptosis decreases as time *t* advances.

Cells undergoing apoptosis take time to fully degrade (62, 63); until apoptosis is completed, the cells still take up space and can inhibit the growth of neighboring cells *in vitro* (37, 63). Upon entering the apoptosis compartment, the time remaining to be spent there is randomly chosen from a probability distribution, e.g., Gamma distribution Γ(*ω*, *λ*) with shape parameter *ω*, rate parameter *λ*, and probability density function *f_A_*(*a*). The choice for this probability distribution is explained in greater detail in Section 2.5.

We noted that the two age-structured PDEs for the proliferative and apoptotic cell compartments enable us to monitor a cell’s progress through the cell cycle (in the case of a cell in *P*) or advancement through apoptosis until complete degradation (in the case of a cell in *A*). Additionally, we assumed that if, upon entering *P*, the time a cell will spend in *P*, *a*, is greater than the threshold $\bar{a}$ (i.e., the cell-cycle age threshold corresponding to a prolonged mitotic arrest), the cell will undergo apoptosis and will thus immediately transition to *A*. This phenomenon has been observed *in vitro* when the sustained prolonged mitotic arrest caused by antimitotic drug exposure leads to apoptotic cell death via the gradual accumulation of cell death signals that ultimately trigger apoptosis. Examples include the phosphorylation and subsequent inactivation of the anti-apoptotic Bcl-2 proteins (Bcl-2, Bcl-xL, and Mcl-1), PARP cleavage, and the activation of caspases 3, 7, and 9 (33, 34, 36–38, 58, 64–66).

### 2.3 Initial Conditions

Initial conditions for the system (1)–(3) are as follows:

(4) P(0,a)=0,

(5) Q(0)=ρ(0)K,

(6) A(0,a)=0.

where *ρ*(0) represents the initial *in vitro* plating density. Here, three different initial conditions are used, i.e., *Q*(0) = 0.1*K*, *Q*(0) = 0.45*K*, and *Q*(0) = 0.8*K*, corresponding to 10, 45, or 80% of the plating carrying capacity *K*, respectively, according to experimental setup in Ref. (48). We noted that the *Q*(0) = 0.1*K* and 0.8*K* cases are identical to the initial conditions reported in Ref. (48). For comparison purposes, we considered in this work, an intermediate case, *Q*(0) = 0.45*K*, which corresponds to the mean value of the two experimental datasets reported in Ref. (48). Therein, the growth dynamics measuring total cellular density every 24 h for a period of 96 h in the two different seeding densities (i.e., 10 and 80% of the *in vitro* plating density) was subsequently recorded. For a more detailed description of the experimental design, we referred to Ref. (48) Appendix B.1.2.

We noted that equation (1) does not require a boundary condition at *a* = 0, since this is a PDE that models a transport process with outward flux only, i.e., once proliferating cells reach *a* = 0, they double, after which both daughter cells return to quiescence before entering another cell cycle.

### 2.4 Inter-Compartmental Dynamics

Following (48), the transition rates that describe the processes of mitotic exit followed by quiescence, mitotic exit, or quiescence followed by the onset of apoptosis are, respectively:

(7) $$\alpha_{\mathrm{QP}}\left(t\right)=c\frac{{\left[\beta \left(\rho \left(t\right)\right)N_{\mathrm{tot}}\left(t\right)-P\left(t\right)\right]}_{+}}{Q\left(t\right)},$$

(8) $$\alpha_{\mathrm{PA}}\left(t\right)=c\gamma \frac{{\left[dN_{\mathrm{tot}}\left(t\right)-A\left(t\right)\right]}_{+}}{P\left(t\right)},$$

(9) $$\alpha_{\mathrm{QA}}\left(t\right)=c\left(1-\gamma \right)\frac{{\left[dN_{\mathrm{tot}}\left(t\right)-A\left(t\right)\right]}_{+}}{Q\left(t\right)}.$$

*P*(*t*), *Q*(*t*), and *A*(*t*) represent the total number of cells at time *t* in the proliferative, quiescent, and apoptotic compartments. Herein, the total number of proliferative and apoptotic cells are integrated over the cellular age, i.e., $\int P\left(t,a\right)\;\mathrm{da}$ and $\int A\left(t,a\right)\;\mathrm{da}$, respectively. The total number of cells that occupy the plate at time *t* is described by *N_tot_*(*t*) = *P*(*t*) + *Q*(*t*) + *A*(*t*). The total number of non-apoptotic cells at time *t* is described by *N*(*t*) = *P*(*t*) + *Q*(*t*). Cell density is denoted by *ρ*(*t*) = *N_tot_*(*t*)/*K*, with *K* representing *in vitro* confluence. Here, *ρ* = 1 when *N_tot_*(*t*) = *K*, which implies that cells have reached confluence at time *t*. For a complete explanation and derivation of the transition rates in (7)–(9), we referred to Ref. (48).

We noted that the functional forms in equations (7)–(9) are time and density dependent and reflect the *in vitro* experimental conditions used in Ref. (48), where OVCAR-8 cells were seeded at different cell densities and initially synchronized to be quiescent using starvation media.

Additionally, we assumed that for a given *in vitro* cell density at time *t*, there exists an equilibrium distribution of cells actively in the cell cycle. This is represented in the model by the function *β*(*ρ*(*t*)), i.e., the fraction of proliferating cells as a function of the *in vitro* cell density *ρ*(*t*) at equilibrium. Experimentally, in order to determine *β*(*ρ*(0)), in Ref. (48), OVCAR-8 human ovarian carcinoma cells seeded at different cell densities were initially synchronized as quiescent, using two distinct cell-cycle arrest experiments performed by changing the starvation media and duration of the experiment. For a more detailed description of the experimental design, we referred to Ref. (48) Appendix B.1.1.

In the model, *β*(*ρ*(*t*)) is described by:

(10) $$\beta \left(\rho \left(t\right)\right)=\beta_{m}e^{\frac{-\theta {\left(\rho -\rho_{m}\right)}^{2}}{\rho {\left(1+\varepsilon -\rho \right)}^{2}}},$$

(11) $$\theta :=\frac{\varepsilon^{2}\text{log}\;\left(\frac{\beta_{m}}{d}\right)}{{\left(1-\rho_{m}\right)}^{2}}.$$

A complete list of the variables and parameters used throughout the modeling framework (1)–(11) and their interpretation can be found in Table 1. We noted that the parameters and functional forms described earlier are adapted from Ref. (48).

**Table 1:** List of variables and parameters used throughout the model.

Variable	Value	Definition
*t*	[0, 200] (hours)	Time
*a*	[0, 80] (hours)	Maximum time remaining to be spent in *P* or *A*
*P*(*t*, *a*)	[0, ∞) (cells)	Number of proliferative cells at time *t* with time *a* to spend in *P*
*Q*(*t*)	[0, ∞) (cells)	Number of quiescent cells at time *t*
*A*(*t*, *a*)	[0, ∞) (cells)	Number of apoptotic cells at time *t* with time *a* to spend in *A*
*N_tot_*(*t*)	[0, ∞) (cells)	Total number of cells at time *t*
*N*(*t*)	[0, ∞) (cells)	Total number of non-apoptotic cells at time *t*
*K*	40,401 (cells)	*In vitro* carrying capacity
*f_P_*(*a*)	[0, ∞)	PDF of 𝒩 (*μ*, *σ*), describing the cell-cycle length without drug
*μ*	[15.23, 19.12] (hours)	Mean cell-cycle length without drug
*σ*	3 (hours)	SD of the cell-cycle length without drug
*f_P,c_*(*a*)	[0, ∞)	PDF of N(μ+c(t),σ), describing the cell-cycle length with drug
*c*(*t*)	[0, ∞) (hours)	Drug-induced mitotic arrest extending the average cell-cycle length
*f_A_*(*a*)	[0, ∞)	PDF of Γ(*ω*, *λ*) describing the length of apoptosis
*ω*	4.9436	Shape parameter for the Gamma-distributed length of apoptosis
*λ*	0.19117	Rate parameter for the Gamma-distributed length of apoptosis
$\bar{a}$	[24.23, 28.12] (hours)	Cell-cycle age threshold corresponding to a prolonged mitotic arrest
*α_QP_*(*t*)	[0, ∞)	Transition rate from *Q* to *P*
*α_PA_*(*t*)	[0, ∞)	Transition rate from *P* to *A*
*α_QA_*(*t*)	[0, ∞)	Transition rate from *Q* to *A*
*c*	[0.37, 0.64] (hour^−1^)	Cellular reaction rate
*γ*	[0.0005, 0.9999]	Transition probability to enter *A*
*ρ*(*t*)	[0, ∞)	*In vitro* cell density at time *t*
*d*	0.03	Fraction of total number of cells in *A*
*β*(*ρ*(*t*))	[0, 1]	Fraction of total number of cells in *P* as a function of *ρ*(*t*)
*β_m_*	[0, 1]	Maximum of *β*(*ρ*(*t*))
*ρ_m_*	[0, 1]	Maximizing density of *β*(*ρ*(*t*))
*ε*	[0, 1]	Parameter governing the shape of *β*(*ρ*(*t*))

### 2.5 Intra-Compartmental Dynamics

The age-structured mathematical model proposed above incorporates an intrinsic form of cell heterogeneity in the *in vitro* cancer cell growth dynamics, specifically in the distribution of times individual cells spend in the cell-cycle and apoptosis process.

To the best of our knowledge, there are no *in vitro* studies describing the distribution of times individual OVCAR-8 cells spend in the cell-cycle. In Ref. (48), Greene et al. chose to model the amount of time OVCAR-8 cells spend in the proliferative compartment, *P*, as a normal distribution, N(μ,σ), with probability density function *f_P_*(*a*). In our model, the density function is re-normalized to integrate to 1 on the interval [0, ∞). Based on the temporal OVCAR-8 growth dynamics reproduced in Figure 4 in Ref. (48), the mean cell-cycle length obtained when fitting to the experimental data is *μ* = 19.12 h, when the initial plating density is set at *Q*(0) = 10% of the maximum plating density, *K*. When the initial plating density is *Q*(0) = 80% of the maximum plating density, *K*, the mean cell-cycle length obtained when fitting to the experimental data is *μ* = 15.23 h. When fitting the system (1)–(4) to the experimental data for both plating density conditions, the mean cell-cycle length obtained is *μ* = 18.33 h. Experimentally, the doubling time reported for OVCAR-8 cells decreases with higher plating density and varies between 14.57 (67) and 26.1 h (68).

The amount of time cells spend in the apoptosis compartment, *A*, is assumed to follow a Gamma distribution, Γ(*ω*, *λ*), where *ω* and *λ* denote the shape and rate parameters, respectively, with probability density function *f_A_*(*a*). These parameters are set at *ω* = 4.9436 and *λ* = 0.19117, respectively, to match the experimental results of Ref. (62) on the length of the apoptotic process. They are identical to the ones used in Ref. (48) to characterize this process. We noted, however, that the study of Ref. (62) investigated the individual responses of PC12 rat adrenal gland tumor cells to serum deprivation. Therein, the authors performed a comprehensive study on the fate of distinct cells undergoing apoptosis following serum removal. To the best of our knowledge, no such studies performed on human cancer cell lines have reported a distribution of the time individual cells spend in apoptosis at such a fine resolution, either in the absence or the presence of antimitotic drugs. We thus chose to model the probability density function of the length of apoptosis process based on the experimental data in Ref. (62). The remaining model parameters listed in Table 1 are obtained following the parameter estimation procedure described in Ref. (48).

### 2.6 Cellular Response to Antimitotic Drugs

In our model, we considered an antimitotic drug whose effect on the cellular dynamics is to induce mitotic arrest, extending the average cell-cycle length. We assumed the administered drug to be homogeneously distributed, such that all cells in *P* are equally susceptible to its effect. Specifically, the impact of the drug is to increase the time cells spend in the proliferative compartment, *P*, corresponding to a sustained mitotic arrest. Upon exiting quiescence and entering the cell cycle, a cell can undergo one of two fates: (i) if the time chosen to be spent in *P* is lower than the threshold $\bar{a}$, the cell enters *P*, progresses through the cell cycle, and either successfully completes mitosis with rate *α_QP_*, or undergoes apoptosis with rate *α_PA_*; (ii) otherwise, the cell commits to undergoing apoptosis and immediately moves to the apoptotic compartment *A*. The parameter $\bar{a}$ serves as the cell-cycle age threshold corresponding to a prolonged mitotic arrest, after which cells exit the cell cycle and undergo apoptosis.

It is a well-known phenomenon *in vitro* that a sustained mitotic arrest (i.e., slowing or blocking of mitosis at the metaphase–anaphase transition, thus increasing cell-cycle length) predisposes cancer cells to undergoing apoptosis following mitotic exit (7, 11, 18, 30, 33, 34, 36–38, 56, 66). This was revealed using time-lapse microscopy data, where exposure of cancer cells to saturating antimitotic drug concentrations delayed to various extents the cells from exiting drug-induced mitotic arrest and undergoing subsequent apoptosis.

In our model formulation, the antimitotic drug acts directly on the cell-cycle dynamics by increasing the average cell-cycle length, and as a consequence, causing cells to transition to the apoptotic compartment. To include the effect of such a drug, we shifted the expected value *μ* of the normal distribution by the function *c*(*t*) corresponding to the cell-length increase induced by the antimitotic drug, i.e., *f_P,c_*(*a*) is the probability density function of the normal distribution $N\left(\mu +c\left(t\right),\sigma \right)$. The system (1)–(12) remains otherwise unchanged. Here, *c*(*t*) can, for example, be modeled as a constant or bang–bang function throughout the duration of the simulation time *t* = 200 h, corresponding to either a sustained, constant mitotic arrest or a switch-on/switch-off arrest.

Experimentally, the sustained, constant mitotic arrest corresponds to the large cell-to-cell variations in the duration of mitotic arrest and the timing of drug-induced cell death via apoptosis observed *in vitro* when single cells are exposed to saturating drug concentrations using various antimitotics for prolonged periods of time, e.g., 96 hours or more (32, 33, 37). We further investigated the impact of an *in silico* switch-on/switch-off mitotic arrest on the overall cancer cell growth dynamics. This type of “bang–bang” mitotic arrest could, for example, be induced *in vitro* by the periodic addition and wash-off of the antimitotic drug under study, along with growth media refreshment. In this setting, when the drug is withdrawn, proliferating cells do not necessarily revert to the cell-cycle length assigned to them in the absence of the drug. Rather, these cells can still undergo a period of mitotic arrest, in which the progression through the cell cycle can be slowed down or blocked, leading to an increase in the cell-cycle length, after which the cell cycle is completed and cells exit proliferation.

We noted that our age-structured modeling framework allows us to estimate the number of cells present in each compartment at any given time and to temporally trace the distribution of the times remaining to be spent in the proliferative phase during the cell cycle or in the apoptotic phase. This framework enables us to dynamically estimate the amount of time remaining to be spent in each of these processes and to track cells in their progression through each cellular phase.

## 3 Results

### 3.1 Cancer Cell Growth Dynamics in the Absence of the Drug

**Figure 2:**
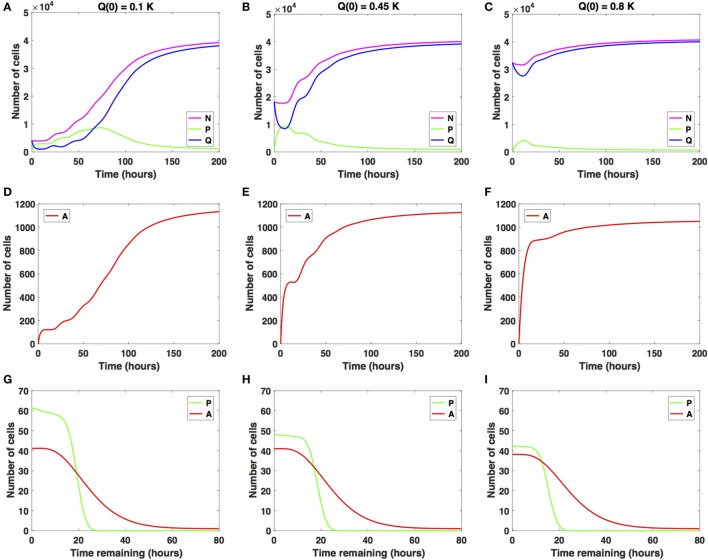
Numerical solutions for the system (1)–(3) in the absence of the drug with **(A,D,G)** *Q*(0) = 10%, **(B,E,H)** *Q*(0) = 45%, and **(C,F,I)** *Q*(0) = 80% of the *in vitro* carrying capacity, *K*. **(A–C)** show the dynamics of the proliferating (*P*), quiescent (*Q*), and total number of non-apoptotic cells (*N*). **(D–F)** show the dynamics of the apoptotic cells (*A*). **(G–I)** illustrate the distribution of the times remaining to be spent by cells in the proliferative (*P*) and apoptotic (*A*) compartments as seen at the end of the simulation time, *t* = 200 h, with **(G)** *Q*(0) = 10%, **(H)** *Q*(0) = 45%, and **(I)** *Q*(0) = 80% of plating carrying capacity, *K*.

We illustrate in Figure 2 the cancer cell growth dynamics modeled by the system (1)–(3), with transition rates (7)–(9) and initial conditions (4)–(6). Specifically, we consider three sets of initial conditions, i.e., *Q*(0) = 0.1*K* in Figures 2A,D,G, *Q*(0) = 0.45*K* in Figures 2A,D,G, and *Q*(0) = 0.8*K* in Figures 2B,E,H, corresponding to 10, 45, or 80% of the plating carrying capacity, *K*, respectively.

The initial plating density, with all cells being experimentally synchronized as quiescent, as described in Section (2.3), substantially alters the overall growth dynamics throughout the simulation time. This can be observed in the relative and absolute numbers of proliferating cells (solid green line) or quiescent cells (solid blue line) and in the total number of cells, i.e., proliferating and quiescent cells (solid magenta line). In the *Q*(0) = 0.1*K* case, the ratio $Q∕P=\frac{Q\left(t\right)}{\int P\left(t,a\right)\;\mathrm{da}}$ (henceforth referred to as *Q*/*P*) is greater than 1 until around *t* = 2 h, after which it becomes smaller than 1 until around *t* = 63 h. Afterward, the ratio *Q*/*P* increases with time. In the *Q*(0) = 0.45*K* case, the ratio *Q*/*P* becomes less than 1 only for a brief period of time, *t* ∈ [7, 13], after which it continues to increase with time. In the *Q*(0) = 0.8*K* case, the number of quiescent cells only decreases for a brief period of time, *t* ∈ [0, 11], after which the number of quiescent cells continues to increase until almost reaching carrying capacity. The ratio *Q*/*P* remains >1 throughout the duration of the simulation.

For comparison purposes, we also illustrate the distribution of the times remaining to be spent in the proliferative (*P*) or apoptotic (*A*) compartment at the end of simulation time (*t* = 200 h), for each of the initial plating densities: *Q*(0) = 0.1*K* in Figure 2G, *Q* = 0.45*K* in Figure 2H, and *Q*(0) = 0.8*K* in Figure 2I. The solid green lines correspond to the distribution of the time remaining to be spent by cells in *P*, and the solid red lines correspond to the times remaining to be spent by cells in *A*.

In each of the three scenarios, all cells are synchronized to be quiescent at the start of the simulation time *t* = 0 h. The long-term dynamics of the system (1)–(3) reveals that the majority of cells are quiescent at the end of the simulation time *t* = 200 h, with *N_tot_*(*t*) close to the carrying capacity. There are few remaining proliferating cells, suggesting that once cells approach confluence, proliferation will be inhibited. The initial plating density does not alter the quantitative nor qualitative dynamics of the apoptotic cell compartment throughout the simulation time (solid red lines). We conclude that at confluence and in the absence of the drug, quiescence is the long-term asymptotic behavior emerging from the cancer cell growth dynamics.

### 3.2 Cancer Cell Growth Dynamics under Antimitotic Drug Action

We now investigate the dynamic behavior of the system (1)–(3) using two distinct antimitotic drug effects, i.e., a sustained, constant mitotic arrest and a switch-on/switch-off arrest, with three different levels of increase in the average cell-cycle length.

In the numerical simulations depicted later, the function *c*(*t*), corresponding to the drug-induced mitotic arrest extending the average cell-cycle length, can take two functional forms: it is set to be a constant function *c*(*t*) = 2*c_arrest_* set at either 2, 10, or 20 h (solid lines) or a bang–bang function *c*(*t*) = 2*c_arrest_* for 0 ≤ *t* ≤ 2 and *c*(*t*) = 0 for 2 ≤ *t* ≤ 4 h, repeated periodically with period 4 until *t* = 200 h (dashed lines).

#### 3.2.1 Cancer Cell Growth Dynamics Given Small Increases in Cell-Cycle Length

We studied the cancer cell growth dynamics given the action of the drug as modeled by the system (1)–(3), with initial conditions (4)–(6). To begin with, we considered small increases in the average cell-cycle length setting *c_arrest_* = 2 h.

**Figure 3:**
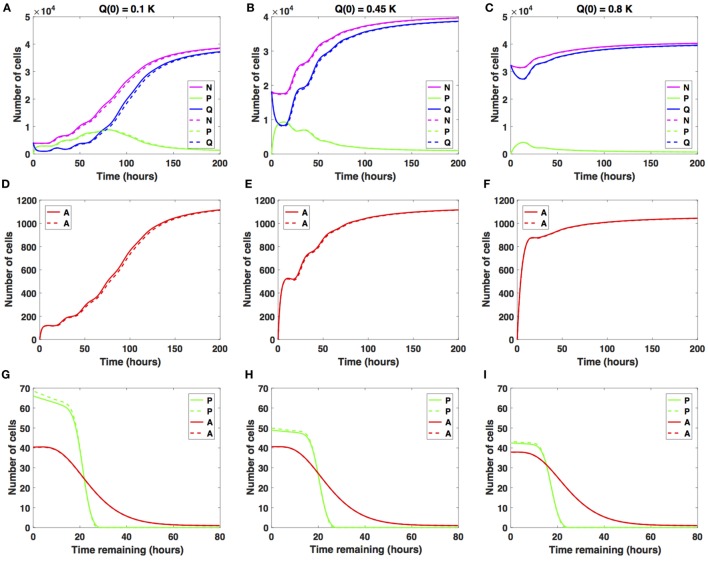
Numerical solutions for the system (1)–(3) given small increases in the average cell-cycle length with **(A,D,G)** *Q*(0) = 10%, **(B,E,H)** *Q*(0) = 45%, and **(C,F,I)** *Q*(0) = 80% of the *in vitro* carrying capacity, *K*. The cellular dynamics in each compartment given a sustained, constant mitotic arrest or a switch-on/switch-off arrest is illustrated using solid or dashed lines, respectively. **(A–C)** show the dynamics of the proliferating (*P*), quiescent (*Q*), and the total number of non-apoptotic cells (*N*). **(D–F)** show the dynamics of the apoptotic cells (*A*). **(G–I)** illustrate the distribution of the times remaining to be spent by cells in the proliferative (*P*) and apoptotic (*A*) compartments as seen at the end of the simulation time, *t* = 200 h, with **(G)** *Q*(0) = 10%, **(H)** *Q*(0) = 45%, and **(I)** *Q*(0) = 80% of plating carrying capacity, *K*.

There is a relatively small difference between the two distinct antimitotic drug effects (see Figure 3, solid versus dashed lines for each color representing the different cellular compartments). Specifically, in both cases, the number of proliferative cells (solid and dashed green lines in Figure 3A) initially increases and then starts to decrease at around *t* = 73 h. The number of quiescent cells (solid and dashed blue lines in Figure 3A) initially decreases and continues to oscillate until around *t* = 40 h, when it begins to increase with time. These oscillations are due to the transitions from *Q* to *P* and back to *Q*. Initially, the ratio *Q*/*P* becomes less than 1 (*t* ∈ [2, 73]), after which it steadily increases beyond 1 throughout the rest of the simulation time. The total number of apoptotic cells integrated over the cellular age, $\int A\left(t,a\right)\;\mathrm{da}$ (solid and dashed red lines in Figure 3D), steadily increases with respect to time. In Figure 3G, we show the distribution of the times remaining to be spent by proliferating cells (green lines) and apoptotic cells (red lines) at the end of simulated time *t* = 200 h, given small increases in average cell-cycle length, using the sustained, constant mitotic arrest (solid lines) and switch-on/switch-off arrest (dashed lines).

The two antimitotic drug effects have no noticeable difference with regard to the cellular dynamics in either of the three compartments. Compared with the cancer cell growth dynamics in the absence of the drug (see Figures 2 and 3), the ratio *Q*/*P* becomes greater than 1 and subsequently increases at a slightly later time point, i.e., at around *t* = 73 versus *t* = 63 h in the absence of the drug.

Similar results are obtained when considering *Q*(0) = 0.45*K* (see Figures 3B,E,H) and when considering *Q*(0) = 0.8*K* (see Figures 3C,F,I). We conclude that nearing confluence and in the presence of small increases in average cell-cycle length, quiescence emerges as the long-term asymptotic behavior resulting from the cancer cell growth dynamics.

#### 3.2.2 Cancer Cell Growth Dynamics Given Intermediate Increases in Cell-Cycle Length

**Figure 4:**
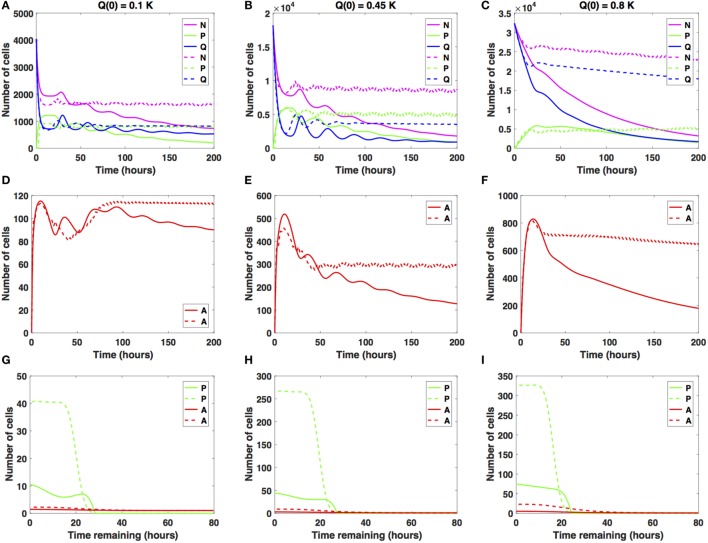
Numerical solutions for the system (1)–(3) given intermediate increases in the average cell-cycle length with **(A,D,G)** *Q*(0) = 10%, **(B,E,H)** *Q*(0) = 45%, and **(C,F,I)** *Q*(0) = 80% of the *in vitro* carrying capacity, *K*. The cellular dynamics in each compartment given a sustained, constant mitotic arrest or a switch-on/switch-off arrest is illustrated using solid or dashed lines, respectively. Panels **(A–C)** show the dynamics of the proliferating (*P*), quiescent (*Q*), and total number of non-apoptotic cells (*N*). **(D–F)** show the dynamics of the apoptotic cells (*A*). **(G–I)** illustrate the distribution of the times remaining to be spent by cells in the proliferative (*P*) and apoptotic (*A*) compartments as seen at the end of the simulation time, *t* = 200 h, with **(G)** *Q*(0) = 10%, **(H)** *Q*(0) = 45%, and **(I)** *Q*(0) = 80% of the plate carrying capacity, *K*.

We now consider intermediate increases in the average cell-cycle length, setting *c_arrest_* = 10 h. Results are shown in Figure 4.

The case *Q*(0) = 0.1*K* is illustrated in Figures 4A,D,G. Specifically, the number of proliferative cells (solid and dashed green lines) fluctuates significantly at the beginning of the numerical simulation for both antimitotic drug effects considered. However, at around *t* = 77.5 h, the number of proliferative cells exposed to the sustained, constant mitotic arrest starts to decrease with time. The number of proliferative cells exposed to the switch-on/switch-off arrest oscillates slightly around the number of quiescent cells.

After the initial decrease in absolute numbers at around *t* = 15 h, the quiescent cells exposed to the sustained, constant mitotic arrest exhibit a pattern of damped oscillations. They continue to slightly decrease in numbers throughout simulation time (solid blue line). The quiescent cells exposed to the switch-on/switch-off arrest seem to have reached a steady state at around *t* = 88 h. Interestingly, for the sustained, constant mitotic arrest, the ratio *Q*/*P* becomes greater than 1 and increases slightly with time starting at around *t* = 78 h. However, for the switch-on/switch-off arrest, the same ratio remains consistently around 1 throughout simulation time, suggesting the existence of a steady-state equilibrium between the proliferative and quiescent populations. A similar pattern can be observed in the dynamics of the total number of proliferating and quiescent cells (solid and dashed magenta lines).

The total number of apoptotic cells (solid and dashed red lines in Figure 4D) oscillates with time. Figure 4G shows the distribution of the times remaining to be spent by proliferating cells (green lines) and by apoptotic cells (red lines) at *t* = 200 h. Similar results are obtained when considering *Q*(0) = 0.45*K* (see Figures 4B,E,H).

However, for *Q*(0) = 0.8*K*, the dynamics of the proliferative (green lines), quiescent (blue lines), and apoptotic (red lines) cell compartments are quantitatively and qualitatively distinct between the two distinct antimitotic drug effects (see Figures 4C,F,I).

Specifically, the number of proliferative cells (solid green line in Figure 4C) in the sustained, constant mitotic arrest case starts to decrease around *t* = 50 h. Given the switch-on/switch-off arrest however, the number of proliferative cells oscillates slightly (dashed green line) starting around *t* = 20 h and continues until the end of the simulated time. The number of quiescent cells (dashed green and blue lines, respectively) continues to steadily decrease for both antimitotic drug effects, with the quiescent cells decaying at a faster rate in the sustained arrest case than in the switch-on/switch-off one (see Figure 4C). A similar pattern can be observed in the dynamics of the total number of cells (proliferating and quiescent), as represented by the solid and dashed magenta lines in Figure 4C. The total number of apoptotic cells (solid and dashed red lines in Figure 4F) starts to decrease at around *t* = 18 h. In Figure 4I, we show the distribution of the times remaining to be spent by proliferating cells (green lines) and apoptotic cells (red lines) at *t* = 200 h.

The two antimitotic drug effects at intermediate increases in cell-cycle length have a marked distinct impact on the cellular dynamics in each of the three cellular compartments for the *Q*(0) = 0.8*K* case. Specifically, the number of quiescent cells decreases in time, and implicitly, the total number of cells decreases at a slower (dashed magenta line) or faster rate (solid magenta line). The dynamics of the cell population illustrated in Figure 4C is overall substantially different from the oscillatory dynamics observed in the *Q*(0) = 0.45*K* and *Q*(0) = 0.1*K* cases. We conclude that in the presence of intermediate increases in the cell-cycle length, the sustained, constant mitotic arrest markedly decreases the total number of cancer cells present. A switch-on/switch-off arrest maintains an active cell population in the long-term, with proliferative cell numbers exhibiting a steady oscillatory state and quiescent cell numbers remaining relatively constant in time.

#### 3.2.3 Cancer Cell Growth Dynamics Given Large Increases in Cell-Cycle Length

**Figure 5:**
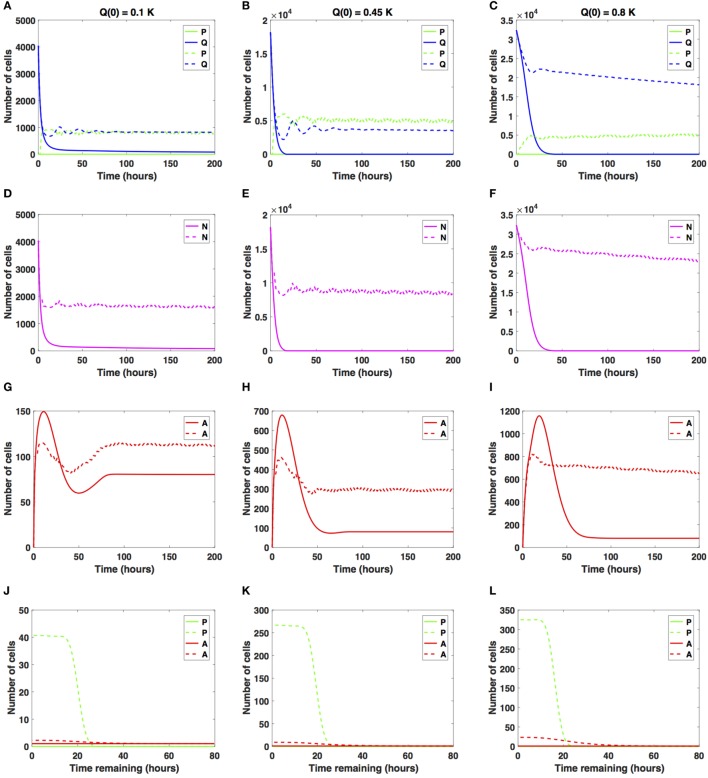
Numerical solutions for the system (1)–(3) given large increases in the average cell-cycle length with **(A,D,G,J)** *Q*(0) = 10%, **(B,E,H,K)** *Q*(0) = 45%, and **(C,F,I,L)** *Q*(0) = 80% of the *in vitro* carrying capacity, *K*. The cellular dynamics in each compartment given a sustained, constant mitotic arrest or a switch-on/switch-off arrest is illustrated using solid or dashed lines, respectively. **(A–F)** show the dynamics of the proliferating (*P*), quiescent (*Q*), and the total number of non-apoptotic cells (*N*). **(G–I)** show the dynamics of the apoptotic cells (*A*). Panels **(J–L)** illustrate the distribution of the times remaining to be spent by cells in the proliferative (*P*) and apoptotic (*A*) compartments as seen at the end of the simulation time, *t* = 200 h, with **(J)** *Q*(0) = 10%, **(K)** *Q*(0) = 45%, and **(L)** *Q*(0) = 80% of the plate carrying capacity, *K*.

We now consider increases in the average cell-cycle length, setting *c_arrest_* = 20 h. Results are shown in Figure 5.

When the initial density is low (*Q*(0) = 0.1*K*), the number of proliferative cells given the sustained, constant mitotic arrest case (solid green line in Figure 5A) remains essentially zero for the entire simulation. Given the large increase in the average cell-cycle length induced by the drug, any cells that transition from *Q* to *P* subsequently transition to *A*, instead of doubling successfully at the end of the cell cycle. However, given the switch-on/switch-off arrest (dashed green line in Figure 5A), proliferative cell numbers exhibit a steady oscillatory state throughout the duration of the simulated time. The ratio *Q*/*P* oscillates around 1 as time increases for the duration of simulation. A similar pattern can be observed in the dynamics of the total number of cells (proliferating and quiescent), as shown by the magenta lines in Figure 5D.

The total number of apoptotic cells (solid and dashed red lines in Figure 5G) oscillates with time. In Figure 5J, we show the distribution of times remaining to be spent by proliferating cells (green lines) and apoptotic cells (red lines) at *t* = 200 h.

Our numerical simulations suggest that in the presence of a sustained, constant mitotic arrest, the cancer cell population is nearly driven to extinction (see solid lines in Figures 5A,D). Intriguingly, in the presence of a long-term switch-on/switch-off arrest, it is possible to maintain an active cancer cell population even when starting with a small initial plating density (*Q*(0) = 0.1*K*) and large increase in the average cell-cycle length. The balance between the quiescent and proliferative cell-turnover is maintained over time (see dashed lines in Figures 5A,D). Similar results are obtained when considering *Q*(0) = 0.45*K*, shown in Figures 5B,E,H,K.

However, when *Q*(0) = 0.8*K*, the dynamics of the proliferative (green lines), quiescent (blue lines), and apoptotic (red lines) cell compartments are quantitatively and qualitatively distinct between the two antimitotic drug effects, with a clear difference between the sustained, constant, and switch-on/switch-off mitotic arrest (see Figures 5C,F,I,L, solid versus dashed lines for each color representing the different cellular compartments).

Specifically, the number of proliferative cells, given the sustained, constant mitotic arrest (solid green line in Figure 5C), remains essentially zero for the entire simulation, similar to the 10 and 45% initial density cases. However, given the switch-on/switch-off mitotic arrest (dashed green line in Figure 5C), proliferative cell numbers exhibit a steady oscillatory state throughout the duration of the simulation. The number of quiescent cells (dashed green and blue lines, respectively) continues to steadily decrease for both drug effects, with quiescent cells decaying at a faster rate in the sustained, constant arrest case than in the switch-on/switch-off one (see Figure 5C). A similar pattern can be observed in the dynamics of the total number of cells (proliferating and quiescent), as represented by the solid and dashed magenta lines in Figure 5F. The total number of apoptotic cells (solid and dashed red lines in Figure 5I) oscillates with time.

Our numerical simulations suggest that in the presence of a large sustained increase in the average cell-cycle length induced by the drug, the cancer cell population is nearly driven to extinction, despite the large initial starting density (see solid lines in Figures 5C,F). Conversely, in the presence of a long-term switch-on/switch-off arrest, it is possible to maintain an active cancer cell population even when starting with a large initial plating density (*Q*(0) = 0.8*K*) and a large increase in the average cell-cycle length. The dynamic balance between the quiescent and proliferative cell turnover is maintained over time (see dashed lines in Figures 5C,F). We conclude that in the presence of large increases in the average cell-cycle length induced by the drug, a sustained, constant mitotic arrest drives both the proliferating and quiescent cell numbers to extinction. A switch-on/switch-off arrest maintains an active cell population in the long-term, with proliferative and quiescent cell numbers exhibiting a steady oscillatory state in time.

## 4 Discussion

The dynamics of cellular response to antimitotic drug exposure has only recently begun to be investigated *in vitro* using time-lapse microscopy on single cells in culture (18, 29, 30, 32–38, 56, 58, 64, 65). Several studies have demonstrated that antimitotic drugs characteristically induce a period of prolonged mitotic arrest (that can last for as long as 72 hours or more) followed predominantly by cell death via apoptosis (32). As such, mitotic arrest constitutes the first cellular response to antimitotic drug exposure, but the mechanisms behind the drug-induced prolonged mitotic arrest and subsequent cancer cell death remain, however, unclear (30–33, 35–37, 64, 65, 69).

To investigate this issue, multiple antimitotic drugs and different drug concentrations have been used in cancer cell studies. Accordingly, multiple *in vitro* single-cell live imaging studies have demonstrated that cancer cells display widely varying responses to antimitotic drugs given different exposure times and drug concentrations (30–33, 35–37, 56, 64, 65, 69). These findings provided strong evidence that the duration of the mitotic arrest is not identical for all cells, both across and within distinct cancer cell lines, in the presence of various antimitotic drugs such as nocodazole, kinesin-5 (Eg5) inhibitors, monastrol, or taxol (29–32, 35, 36).

Even within identical types of cell cultures or drugs used, cells exhibit a considerable degree of heterogeneity in response to prolonged antimitotic drug exposure. For example, cells may either exit mitosis and remain in interphase for an indefinite period of time, undergo programmed cell death (i.e., apoptosis) after exiting mitosis or interphase, or proceed through mitosis via multipolar spindle formation (29, 31–33, 35–37, 69). In the case of multipolar spindle formation, cells divide into daughter cells by segregating their chromosomes in more than two different directions, dying during the second mitosis, or remaining in interphase for the duration of the experiments (33, 69, 70).

Motivated by these experimental findings, we introduce a novel mathematical modeling framework of cancer cell dynamics given drug exposure that incorporates an intrinsic form of heterogeneity in response to prolonged antimitotic drug exposure via the duration of times cells spend in the cell cycle and apoptosis process. The system (1)–(3) is an age-structured, physiologically motivated modeling framework for describing *in vitro* cancer cell growth dynamics given a drug that induces mitotic arrest, thus extending the average cell-cycle length. To reflect the intrinsic cell heterogeneity, cells in the proliferative and in the apoptotic compartment are structured by the amount of time they spend in each phase. Herein, we considered a drug that extends the average cell-cycle length and studied its impact on the long-term cancer cell growth dynamics and response to antimitotic drug exposure using two distinct antimitotic drug effects, i.e., a sustained, constant mitotic arrest and a switch-on/switch-off arrest and three different levels of increase in the average cell-cycle lengths.

Our numerical simulations suggest that at confluence and in the absence of any drug, quiescence is the long-term asymptotic behavior emerging from the cancer cell growth dynamics. Upon drug addition, the cancer cell dynamics significantly changes. Specifically, the prolonged mitotic arrest induced by the antimitotic drug results in a strong growth-inhibitory activity *in vitro* in a time-dependent manner. In the presence of small increases in the average cell-cycle length, quiescence emerges as the long-term asymptotic behavior resulting from the cancer cell growth dynamics. Our numerical simulations suggest that quiescence can emerge relatively quickly and can thus constitute an intrinsic resistance mechanism to antimitotic drug exposure. The small increases in the average cell-cycle length result in a period of slowing down of the cell cycle from which cancer cells can recover and continue proliferating until reaching confluence. From a therapeutic point of view, the presence of quiescent cancer cells has serious implications for chemotherapy regimens, which rely on active cell cycling to target and kill proliferating cells. The long-term maintenance of a quiescent cancer cell population acts as a reservoir for proliferating cells and can ultimately lead to cancer recurrence and shorter disease-free survival periods (7–9, 71, 72).

However, in the presence of intermediate increases in the average cell-cycle length, a sustained, constant mitotic arrest markedly decreases the total number of cancer cells present and can drive the cell population to extinction. A switch-on/switch-off arrest maintains an active cell population in the long term, with proliferative cell numbers exhibiting a steady oscillatory state and quiescent cell numbers remaining relatively constant in time. The transient behavior in the cancer cell growth dynamics signals the emergence and maintenance of a steady quiescent cell population, which in turn represents a form of intrinsic, non-genetic resistance that results from variations in cell-cycle parameters (73, 74). This can potentially decrease the efficacy of therapies that rely on active cell cycling for their killing effects, such as traditional chemotherapies (75–77). Moreover, given large increases in the average cell-cycle length induced by antimitotics, cells do not resume proliferation and are driven to extinction by a sustained, constant mitotic arrest. Intriguingly, a switch-on/switch-off arrest may maintain an active cancer cell population in the long term. This suggests that unless exposed to saturating drug concentrations for prolonged periods of time, cancer cells may not experience a mitotic arrest for long enough in order to trigger apoptosis, which may have therapeutic implications as clinical responses depend on apoptosis rates and not exclusively on mitotic arrest (18, 69).

Additionally, the fate of cells following drug treatment also depends on the cell type. For instance, cell lines sensitive to mitotic cell death tend to reach the MOMP threshold before cyclin B1 levels reach the threshold required for cells to slip out of mitosis (29, 32, 33, 35, 37, 69). Conversely, cell lines resistant to mitotic cell death tend to have a faster rate of cyclin B1 degradation and/or slow rate of intrinsic cell death activation (34, 36, 38, 58). These molecular-based variations in sensitivity to apoptosis and mitotic arrest are likely to substantially contribute to the observed heterogeneity in cell responses and potentially represent the crucial factor in determining cell fate in response to antimitotic drug exposure.

## Author Contributions

Conceived and designed the experiments: AL, D-AB, and DL. Acquired, analyzed, and interpreted the data: AL, D-AB, and DL. Drafted the manuscript: AL, D-AB, and DL. Approved the final version of the manuscript: AL, D-AB, and DL.

## Conflict of Interest Statement

The authors declare that the research was conducted in the absence of any commercial or financial relationships that could be construed as a potential conflict of interest.
